# Detecting the Influence of Spreading in Social Networks with Excitable Sensor Networks

**DOI:** 10.1371/journal.pone.0124848

**Published:** 2015-05-07

**Authors:** Sen Pei, Shaoting Tang, Zhiming Zheng

**Affiliations:** 1 School of Mathematics and Systems Science, Beihang University, Beijing, China; 2 Laboratory of Mathematics, Informatics and Behavioral Semantics, Ministry of Education, Beijing, China; Hangzhou Normal University, CHINA

## Abstract

Detecting spreading outbreaks in social networks with sensors is of great significance in applications. Inspired by the formation mechanism of humans’ physical sensations to external stimuli, we propose a new method to detect the influence of spreading by constructing excitable sensor networks. Exploiting the amplifying effect of excitable sensor networks, our method can better detect small-scale spreading processes. At the same time, it can also distinguish large-scale diffusion instances due to the self-inhibition effect of excitable elements. Through simulations of diverse spreading dynamics on typical real-world social networks (Facebook, coauthor, and email social networks), we find that the excitable sensor networks are capable of detecting and ranking spreading processes in a much wider range of influence than other commonly used sensor placement methods, such as random, targeted, acquaintance and distance strategies. In addition, we validate the efficacy of our method with diffusion data from a real-world online social system, Twitter. We find that our method can detect more spreading topics in practice. Our approach provides a new direction in spreading detection and should be useful for designing effective detection methods.

## Introduction

Plenty of phenomena in various domains can be depicted by the spreading dynamics in social networks, e.g., the outbreak of a contagious disease [1–4], the diffusion of a piece of information [5–8], or the promotion of a commercial product [9–11]. The detection of spreading processes in social networks is an important issue in many real-world applications, such as the formulation of timely intervention measures during the spread of an epidemic, and the surveillance of current trending topics in popular online social networks. In recent years, the global outbreaks of seasonal influenza and the widespread use of online social media in political movements are reminiscent of the vital role of spreading detection [12]. Because of its practical value, designing effective detection methods has attracted much attention across disciplines. In particular, several Internet-based surveillance systems for disease outbreaks have been proposed [13–16]. By monitoring health-seeking behaviors in the form of online search engine queries or analyzing symptom-related terms appearing in online social media, these systems can estimate the current level of spreading activity. Another approach is to deploy social network sensors in the system. A heuristic algorithm for the optimal placement of sensors has been proposed for spreading models [17], and it has been shown that properly placed sensors can detect contagious outbreaks before they happen in large scale by taking advantage of the informative properties of social networks [18]. Moreover, recent studies indicate that the origin of a spreading process can be inferred by placing sensors in social networks [19, 20]. In addition, there exist extensive studies on predicting the evolution of spreading from a snapshot [21–23]. All these approaches make contributions to the detection and prediction of spreading processes in social networks.

Most of the existing detection methods focus on the objective of early detection. However, considering the large numbers of spreading occurring simultaneously in social networks, it is also important to detect and distinguish the influence of these spreading processes. In specific, our goal is to detect as many spreading processes as possible and rank their relative influence at the same time. This enables us to have an estimation of these spreading processes, which can be applied to applications such as selecting and ranking blogs in a blog community. To achieve this goal, we develop an alternative approach by making use of excitable sensors. In the field of psychophysics, it has been well established that the cooperative effect of excitable elements can be used to explain how physical stimuli (sound, light and pressure) transduce into psychological sensations [24]. Although each single excitable node responds to stimuli with a small range, the collective response of the entire excitable system can encode stimuli spanning several orders of magnitude, yielding high sensitivity and a broad dynamic range (the range of stimulus intensities resulting in distinguishable network responses) [24, 25]. Borrowing the property of nonlinear amplification of stimuli, the excitable elements are suitable for detecting the influence of spreading in social networks: the stimuli can be regarded as infections, and the response of the excitable system can be treated as the detected influence.

Following this idea, we propose a method to deploy excitable sensor networks in social systems. For both homogeneous and heterogeneous networks, we analytically derive the relationship between the response and spreading influence. Through simulations on Erdös-Rényi (ER) and Barabási-Albert (BA) networks, we verify our theoretical analysis for susceptible-infected-recovered (SIR) model, susceptible-infected-susceptible (SIS) model, rumor spreading (Rumor) model and susceptible-infected-recovered model with limited contacting ability (SIRL) [26–30]. Then, we compare the performance of excitable sensor networks with several commonly used sensor placement strategies. The simulation results of spreading models on three typical social networks (including facebook, coauthor and email social networks) suggest that excitable sensor networks outperform the other considered methods: excitable sensor networks can not only grasp small-scale spreading processes because of the amplifying effect of collective dynamics, but also better distinguish large-scale diffusion instances due to the self-inhibition effect of excitable elements. Under the same circumstances, our method has a larger dynamic range, which means it can detect spreading in a wider range of influence. In addition, we discuss the impact of the construction method of excitable sensor networks. We find that the homogeneous sensor networks perform better than heterogeneous ones, and the choice of sensors’ number will not affect our results significantly. We also explore spreading processes originating from different sources. The amplifying effect of excitable sensors is found to be more effective for well-connected sources. Therefore, excitable sensor networks are more likely to detect spreading instances from high-degree sources. Furthermore, we validate the efficacy of our method using real diffusion data from Twitter. We track the spreading of 309 topics in Twitter among selected users, and use each method to detect them. The results show that the excitable sensor network can detect more spreading topics than other strategies under the same situations.

## Materials and Methods

### Datasets

In the numerical simulations, we utilize three different typical social networks as the substrates on which spreading processes occurs. Here we introduce the properties and statistics of each adopted social network. Notice that we treat all the networks as undirected.

**Facebook** The social network of facebook contains all of the user-to-user links from the Facebook New Orleans networks [31]. If one user is in the friend list of another user, then an undirected social link is constructed between them. It contains 63731 nodes and 1545685 links, resulting in an average degree of 48.5. This dataset is shared at http://socialnetworks.mpi-sws.org/data-wosn2009.html.

**Coauthor** Based on the DBLP computer science bibliography which provides a comprehensive list of research papers in computer science, a coauthorship network is constructed [32]. In the network, two authors are connected if they publish at least one paper together. There are 317080 nodes and 1049866 links in the network, and the average degree is 6.7. Researchers can download this dataset at http://snap.stanford.edu/data/com-DBLP.html.

**Email** In the Enron email communication network [33], nodes are email addresses. If an address *i* sends at least one email to another address *j*, the graph contains an undirected edge connecting *i* to *j*. The network contains 36692 nodes and 183831 links, and the average degree is 10.0. The data source is http://snap.stanford.edu/data/email-Enron.html.

In the validation of excitable sensor networks, we use real diffusion data from the microblog platform Twitter. The tweets are sampled between January 23rd and February 8th, 2011 and are shared by Twitter (http://trec.nist.gov/data/tweets/). In Twitter, specific topics are usually labelled by a “hashtag”, i.e., a word or an unspaced phrase prefixed with the number sign (“#”). Therefore, we can use hashtags to track the spreading of a specific topic in Twitter. During the collection period, there happened to be a mass protest on January 25th in Egypt, which was an important event of the “Arab Spring”. In this event, Twitter was used by protesters to organize the protest and recruit members. Many Twitter users discussed and shared information about this protest using Twitter. The most used hashtag related to this protest is “#Jan25”. To obtain the information spreading among users participating in this protest, we filtered 23712 tweets containing “#Jan25” published by 7014 different users. Then, we checked all the available tweets that were published by these users and found 309 distinct topics appearing more than 10 times. To reconstruct the social network of the selected users, we have extracted the mention network, where two users are connected if one user has mentioned another user (“@username”) at least once. Compared with the follower network, the mention network stands for stronger social relations because mentions usually contain personal communications. In this way, we obtain 10547 social links among 7014 users, and the average degree is 3.0.

### Spreading Models

In this paper, we have applied four spreading models to verify the effectiveness of excitable sensor networks. These models were frequently used in previous research works on spreading dynamics. We introduce the details of each model here.

**SIR model** The SIR model is suitable to describe the spreading of a disease with immunity. In the SIR model, each individual is in one of three states: the susceptible (S), infected (I) and recovered (R). At each time step, infected nodes infect their susceptible neighbors with probability *β* and then enter the recovered state with probability *μ*, where they become immunized and cannot be infected again. When there are no more infected individuals in the system, the fraction of recovered person, or equivalently, the fraction of people who have ever been infected, is denoted by *M*. Assume the densities of the susceptible, infected, and recovered at time *t* are *s*(*t*), *i*(*t*), and *r*(*t*) respectively. In homogeneous random networks with average degree ⟨*k*⟩, the dynamics of SIR model satisfies the following set of coupled differential equations

$$\begin{array}{c} \left\{ \begin{array}{c} \frac{di(t)}{dt}=\beta \left\langle k\right\rangle s\left(t\right)i\left(t\right)-\mu i\left(t\right),\\ \frac{ds(t)}{dt}=-\beta \left\langle k\right\rangle s\left(t\right)i\left(t\right),\\ \frac{dr(t)}{dt}=\mu i\left(t\right). \end{array}\right. \end{array}$$ (1)

**SIS model** The SIS model, in which only two states, susceptible (S) and infected (I), are considered, describes spreading processes that do not confer immunity on recovered individuals. In the spreading process, infected individuals infect their susceptible neighbors with probability *β* and return to S state with probability *μ*. As time evolves, the fraction of infected persons *ρ* will become steady. We run SIS dynamics for 100 steps and take the average infected proportion of last 30 steps as *ρ*. In ER random networks with average degree ⟨*k*⟩, the dynamics can be described by the differential equation

$$\begin{array}{c} \frac{di(t)}{dt}=\beta \left\langle k\right\rangle i\left(t\right)\left(1-i\left(t\right)\right)-\mu i\left(t\right). \end{array}$$ (2)

**Rumor model** In Rumor model, each individual can be in three possible states: the spreader (S), ignorant (I), and stifler (R). Spreaders represent nodes that are aware of the rumor and are willing to transmit it. Ignorant people are individuals unaware of the rumor. Stiflers stand for those that already know the rumor but are not willing to spread it anymore. In each time step, the spreaders contact all their neighbors and turn the ignorant ones into spreaders with probability *β*. If the spreaders encounter spreaders or stiflers, they will turn to stiflers with probability *μ*. The influence of the rumor *M* is defined as the fraction of stiflers when there are no more spreaders in the system. In homogeneous networks with average degree ⟨*k*⟩, dynamics follows the set of coupled differential equations

$$\begin{array}{c} \left\{ \begin{array}{c} \frac{di(t)}{dt}=-\beta \left\langle k\right\rangle i\left(t\right)s\left(t\right),\\ \frac{ds(t)}{dt}=\beta \left\langle k\right\rangle i\left(t\right)s\left(t\right)-\mu \left\langle k\right\rangle s\left(t\right)\left[s\left(t\right)+r\left(t\right)\right],\\ \frac{dr(t)}{dt}=\mu \left\langle k\right\rangle s\left(t\right)\left[s\left(t\right)+r\left(t\right)\right]. \end{array}\right. \end{array}$$ (3)

**SIRL model** The SIRL model is a modified SIR model, in which each node is assigned with an identical capability of active contacts, *L*. It stands for the type of spreading with limited contacting ability. Compared with the standard SIR model, at each time step in SIRL model, each infected individual will generate *L* contacts. Multiple contacts to one neighbor are allowed, and contacts not between susceptible and infected ones are also counted just like the standard SIR model. The parameters *β* and *μ* represent the infection rate and recover rate respectively. In homogeneous random networks with average degree ⟨*k*⟩, the dynamics can be calculated by the set of differential equations

$$\begin{array}{c} \left\{ \begin{array}{c} \frac{di(t)}{dt}=\beta Ls\left(t\right)i\left(t\right)-\mu i\left(t\right),\\ \frac{ds(t)}{dt}=-\beta Ls\left(t\right)i\left(t\right),\\ \frac{dr(t)}{dt}=\mu i\left(t\right). \end{array}\right. \end{array}$$ (4)

## Results

### Construction of the excitable sensor network

**Fig 1:**
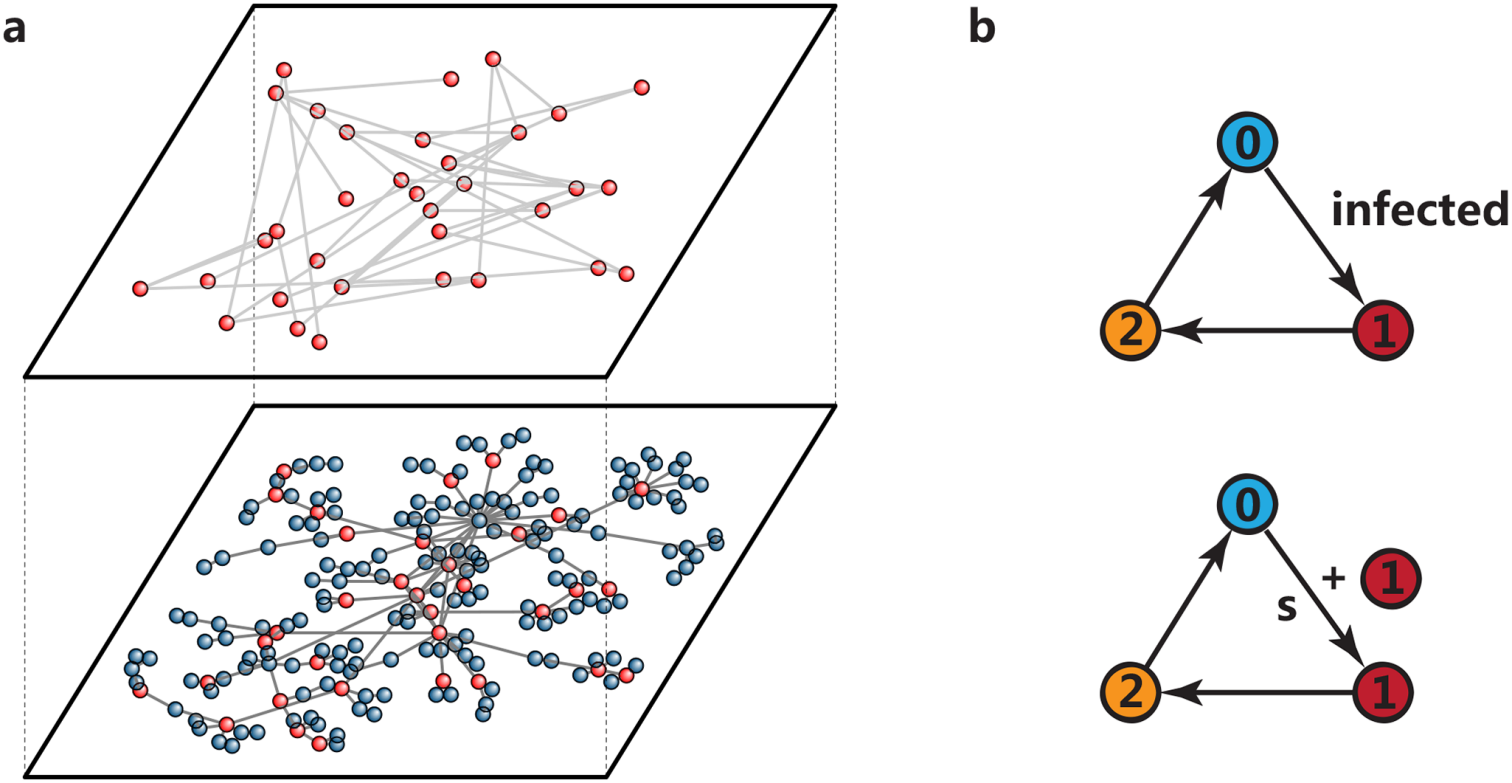
Illustrations of excitable sensor networks and the dynamics of sensors. (a) A schema of a sensor network. The lower layer is the underlying social network and the upper layer represents the sensor network. Both blue and red balls are individuals in the social network and red balls are selected to be sensors. The spreading dynamics and signal transmission occur in the lower and upper level separately. (b) The dynamics of excitable sensors. The number 0, 1 and 2 stand for the resting, excitation and refractory state, respectively. Each sensor in resting state can be activated either by means of infection in the spreading dynamics or by excited neighboring sensors with probability *s* independently. Once activated, the sensors will automatically turn into the refractory state in the next time step, where they cannot be activated again and activate other sensors. Then, these sensors change back to resting state.

We now describe how to construct an excitable sensor network in a social network. Each excitable node has *n* states: *s*_*i*_ = 0 is the resting state, *s*_*i*_ = 1 corresponds to excitation, and the remaining *s*_*i*_ = 2, …, *n* − 1 are refractory states. In our study, we set *n* = 3. Given a social network, we select *f* percent of nodes as sensors according to specific criteria. Then, we create links between the selected sensors to form a network. In our case, we construct a homogeneous random network among selected sensors. Assuming there are *N*_*s*_ nodes in the sensor network, we assign *N*_*s*_⟨*k*⟩/2 links to randomly chosen pairs of nodes, which produces an average degree ⟨*k*⟩. Fig 1(a) illustrates an instance of a sensor network. The lower layer is the underlying social network, while the upper layer is the sensor network.

There are two dynamical processes in the system: spreading dynamics in the social network and signal transmission dynamics in the sensor network. In our study, we adopt four spreading models that are frequently used in the research of spreading dynamics: SIR, SIS, Rumor and SIRL models [26–30]. The SIR model is developed to describe the contagion process of diseases with immunity. Once people recover from the disease, they will acquire permanent immunity. The SIS model depicts the outbreaks of contagions that an individual can catch more than once. The Rumor model considers the diffusion process of rumors among a population. The SIRL model is a modified SIR model, in which each node has a limited capability of active contacts *L* at each time step. The individual-level spreading mechanisms in these models confine the diffusion processes within the underlying social networks, which makes them suitable for exploring the interplay between the spreading dynamics and social network structures. Since the early days of the research of complex networks, these models have been widely employed to simulate the epidemic spreading of different types of diseases [3, 29, 30, 34–36], information diffusion among individuals [27, 37–39], and rumor propagation in online social networks [28, 40]. The details of these models are explained in the Materials and Methods section.

In the following analysis, if an individual catches a contagious disease or becomes aware of a piece of rumor, we define its state as infected. For SIR, Rumor and SIRL models, the influence of a spreading instance *M* is defined as the proportion of people who have ever been infected. In contrast, in SIS model, because people can be infected repeatedly, we define the influence *ρ* as the fraction of infected persons when the system reaches a steady state.

Signal transmission dynamics occur in sensor networks. The evolution dynamics of sensors are shown in Fig 1(b). At each time step, sensors in resting state can evolve into active state under two circumstances: infected in spreading dynamics or activated by active neighboring sensors with probability *s* (coupling strength). The active sensors will update into refractory state and then change back into resting state deterministically. In refractory state, sensors can neither be activated nor activate other neighbors. To define the response of a sensor network to the spreading process, we assume that the observation time is *T*. Denote the activity level of sensors *F* ^*t*^ at time *t* as the proportion of active sensors. The response *F* is defined as the average activity level during the observation time, $F=\sum_{t=0}^{T}F^{t}/T$.

For a spreading process with influence *M*, the system will feedback a response *F*. As a function of the influence *M*, response has a minimum value *F*_0_ and a maximum value *F*_*max*_. To quantify the ability of excitable sensor networks to detect spreading, we define the dynamic range Δ = 10log_10_(*M*_*high*_/*M*_*low*_) as the range of influence that is distinguishable based on response *F*, discarding diffusion processes that are too small to be distinguished from *F*_0_ or that are too close to saturation [24]. The range [*M*_*low*_, *M*_*high*_] is found from its corresponding response interval [*F*_*low*_, *F*_*high*_], where *F*_*x*_ = *F*_0_ + *x*(*F*_*max*_ − *F*_0_).

The dynamics of excitable sensor networks are highly related to the network topology and coupling strength *s*. Many previous studies have shown that there exists a critical point in excitable networks. Only above the critical point, does self-sustained activity emerge in response to external stimuli. It has been proved that the critical state occurs when the largest eigenvalue of the interacting adjacency matrix is exactly 1 [25]. Particularly, a network of excitable elements has its sensitivity and dynamic range maximized at the critical point [25, 41–43]. Therefore, we select the coupling strength *s* to make the sensor network achieve a critical state. For random networks with average degree ⟨*k*⟩ and coupling strength *s*, the largest eigenvalue of the interacting adjacency matrix is approximated by *s*⟨*k*⟩. Thus, in designing the excitable sensor networks, we set the coupling strength *s* = 1/⟨*k*⟩ to optimize the dynamic range.

### Dynamics of excitable sensors

The evolution dynamics of excitable sensors can be studied through theoretical analysis. For each sensor *i* = 1, ⋯, *N*_*s*_, we denote the probability in active state at time *t* as $p_{i}^{t}$. Then, the activity level of sensors is $F^{t}=\sum_{i=1}^{N_{s}}p_{i}^{t}/N_{s}$. In the case of small-scale spreading, we assume that sensors are activated independently by their neighboring active sensors. Therefore, the evolution of $p_{i}^{t}$ follows

$$\begin{array}{c} p_{i}^{t+1}=\left(1-p_{i}^{t}-p_{i}^{t-1}\right)\left(I_{i}^{t}+\left(1-I_{i}^{t}\right)\left[1-\prod_{j=1}^{N_{s}}\left(1-p_{j}^{t}sA_{ij}\right)\right]\right), \end{array}$$ (5)

where $I_{i}^{t}$ is the probability of sensor *i* being activated by infection at time *t*, *s* is the coupling strength of sensors, and *A* is the adjacency matrix of the sensor network: *A*_*ij*_ = 1 if sensor *i* and *j* are connected, and *A*_*ij*_ = 0 otherwise. The term $\left(1-p_{i}^{t}-p_{i}^{t-1}\right)$ is the probability that sensor *i* is at resting state at time *t*, while the term $\left(1-I_{i}^{t})[1-\prod_{j=1}^{N_{s}}(1-p_{j}^{t}sA_{ij})\right]$ is the probability of sensor *i* being activated by its neighboring sensors at time *t*, rather than by infection.

To solve Eq 5, we need to know the infection intensity $I_{i}^{t}$. However, $I_{i}^{t}$ is highly related to sensor *i*’s topological property. For instance, hubs are more likely to be infected [35, 44]. To eliminate the influence of topological structure, we first adopt ER random networks in simulations, and select sensors randomly. Under this condition, we suppose the infection intensity $I_{i}^{t}$ for each sensor *i* is approximately the same. Because the sensor network is homogeneous, we assume that the active probability $p_{i}^{t}$ is approximately the same for all the sensors, i.e., $p_{1}^{t}\approx \cdots \approx p_{N_{s}}^{t}\approx F^{t}$. In addition, using mean-field approximation, the term $\prod_{j=1}^{N_{s}}(1-p_{j}^{t}sA_{ij})$ can be estimated by (1 − *F* ^*t*^ *s*)^⟨*k*⟩^. Consequently, Eq 5 is simplified to

$$\begin{array}{c} F^{t+1}=\left(1-F^{t}-F^{t-1}\right)\left(I^{t}+\left(1-I^{t}\right)\left[1-{\left(1-F^{t}s\right)}^{\left\langle k\right\rangle }\right]\right). \end{array}$$ (6)

Because the sensor network is at the critical state, we have ⟨*k*⟩*s* = 1. For small-scale spreading, *F* ^*t*^ is close to zero. To second order, the term [1 − (1 − *F* ^*t*^ *s*)^⟨*k*⟩^] can be approximated by *F* ^*t*^ + (⟨*k*⟩ − 1)(*F* ^*t*^)^2^/2⟨*k*⟩. Then, Eq 6 is further reduced to

$$\begin{array}{c} F^{t+1}=I^{t}+\left(1-2I^{t}\right)F^{t}-I^{t}F^{t-1}-C\left(1-I^{t}\right){\left(F^{t}\right)}^{2}-\left(1-I^{t}\right)F^{t}F^{t-1}, \end{array}$$ (7)

where *C* = (3⟨*k*⟩ − 1)/2⟨*k*⟩.

Eq 7 is an iterative updating function of *F* ^*t*^. In fact, *F* ^*t*+1^ depends on *F* ^*t*^, *F* ^*t*−1^ and *I* ^*t*^. The infection intensity *I* ^*t*^ is governed by the spreading dynamics. In homogeneous networks, the evolution of *I* ^*t*^ satisfies specific differential equations for SIR, SIS, Rumor and SIRL models, as shown in the Materials and Methods section. By solving these equations, we can calculate the infection intensity *I* ^*t*^. For SIR, SIS and SIRL spreading, *I* ^*t*^ is the proportion of infected people *i*(*t*). For Rumor model, *I* ^*t*^ is the density of spreaders *s*(*t*). With this information, we calculate the response of the sensor network by using the initial conditions *F* ^0^ = 0, *F* ^1^ = *I* ^0^. In the cases of SIR, Rumor and SIRL models, iterations continue until *I* ^*t*^ becomes zero. For SIS model, iteration stops after *I* ^*t*^ becomes steady. The influence of spreading can also be obtained by these equations. For SIR, Rumor and SIRL models, the influence *M* is the value of *r*(*t*) when there are no infected people or spreaders. The influence *ρ* for SIS model is the value of *i*(*t*) in the steady state.

**Fig 2:**
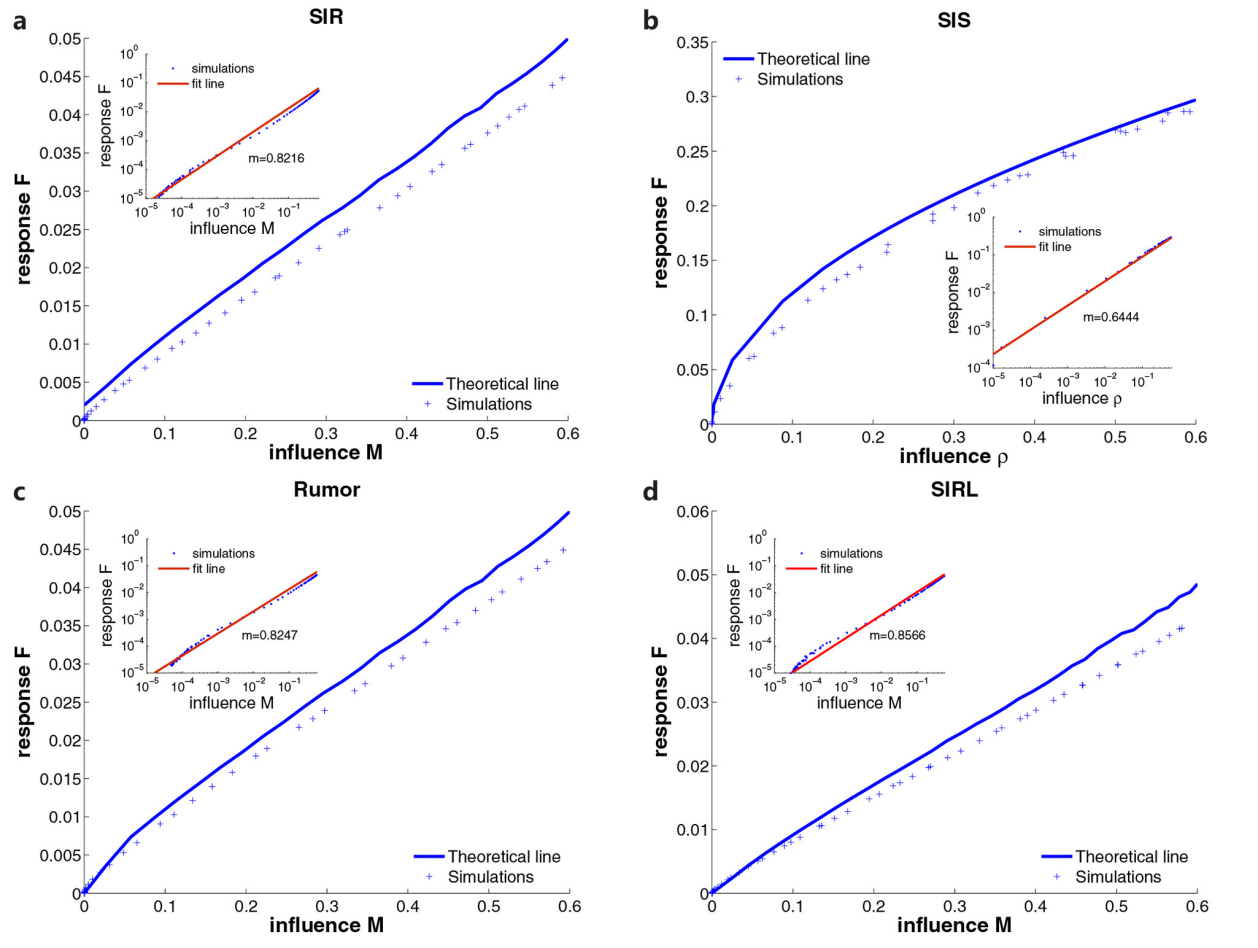
Theoretical analysis of the dynamics of excitable sensors. For SIR, SIS, Rumor and SIRL models, we display the relationship between the response and influence in (a), (b), (c) and (d) respectively. We adopt ER random networks with size 10^5^ and average degree 10. 10% of nodes are randomly selected to be sensors, which are connected in a homogeneous random network with average degree ⟨*k*⟩ = 10. Solid lines are theoretical predictions and cross symbols represent simulation values. In simulations, we vary infection rate *β* and keep *μ* = 1 for all models. The contacting ability *L* in SIRL model is set to be 5. Insets show the power-law fit of the data, where *m* is the power-law exponent.

To verify our theoretical analysis, we run simulations on ER random networks. We generate ER random networks with size 10^5^ and average degree 10. Then, we randomly choose 10% of nodes as sensors, forming a random network with average degree ⟨*k*⟩ = 10. In each simulation, we randomly select a node as the spreading source. For each spreading model, we calculate the theoretical values of response and influence for different infection rates *β* as we have explained above. In Fig 2, the theoretical lines agree well with the simulation results for all considered spreading dynamics. Moreover, we find that the response *F* follows a power-law relation with influence, which is displayed in the insets of Fig 2. Clearly, the power-law exponent *m* depends on spreading dynamics.

In terms of heterogeneous networks, we need to calculate $I_{i}^{t}$ for each sensor *i*. Given the adjacency matrix $\bar{A}$ of the social network, one can obtain $I_{i}^{t}$ for each spreading model by iterating corresponding evolution equations, which are given in Supporting Information S1 File. Combining the information of $I_{i}^{t}$ and Eq 5, we are able to calculate the theoretical values of response and influence. In simulations, we generate BA scale-free networks with size 10^5^ and average degree 10 [45]. The simulation results and theoretical values are presented in Figure A in S1 File. The analytical analysis can well predict simulation results.

### Performance on spreading models

There are several commonly used strategies to place sensors in social networks. We introduce four of them to test the efficacy of excitable sensor networks. The most straightforward method is to randomly select nodes as detecting sensors. Another heuristic alternative is to select hubs as sensors. It has been shown that hubs are easily infected during an outbreak because they possess large numbers of connections to other nodes [35, 44]. Therefore, we can pick nodes with degree ranking in top *f* percent as sensors to catch spreading instances of small influence. The third method is to monitor the friends of randomly selected individuals [18]. It is known that individuals located in the center of networks are more likely to be infected [37, 46]. Generally speaking, the neighbors of a randomly chosen person tend to have larger number of connections and higher *k*-shell indices [18]. Moreover, this strategy is applicable even when we lack complete information about the topological structures. Apart from these sensor placing strategies, another important approach is based on the distance centrality or the Jordan center of a graph [19, 47–49]. For a graph *G*, the distance centrality of node *i* ∈ *G*, *D*(*i*, *G*), is defined as *D*(*i*, *G*) = ∑_*j*∈*G*_ *d*(*i*, *j*), where *d*(*i*, *j*) is the shortest path distance from node *i* to node *j*[49]. Intuitively, nodes with smaller distance centrality are closer to other nodes. Consequently, nodes with small distance centrality are selected as sensors in this strategy. After placing the sensors, we monitor the statuses of these sensors during a spreading process. The influence of spreading is detected as the proportion of infected sensors in the cases of SIR, Rumor and SIRL models or the fraction of sustained infection in the steady state for SIS model. In the following analysis, we refer to these four strategies as *random*, *targeted*, *acquaintance* and *distance* methods, respectively.

To compare the efficacy of excitable sensor networks with other methods, we apply spreading dynamics on three different types of real-world social networks: an online social network—facebook [31], a coauthorship network of scientific publications [32], and a communication network of emails [33]. Explanations and details of the networks can be found in the Materials and Methods section. The selected networks are representative in their corresponding domains, and are widely used in previous studies of social networks. Therefore, these networks can reflect the characteristics of social networks in real life. In simulations, because nodes with more connections have a larger chance to be infected [35, 44], we select nodes ranking in top *f* percent in degree as sensors in the excitable sensor network.

**Fig 3:**
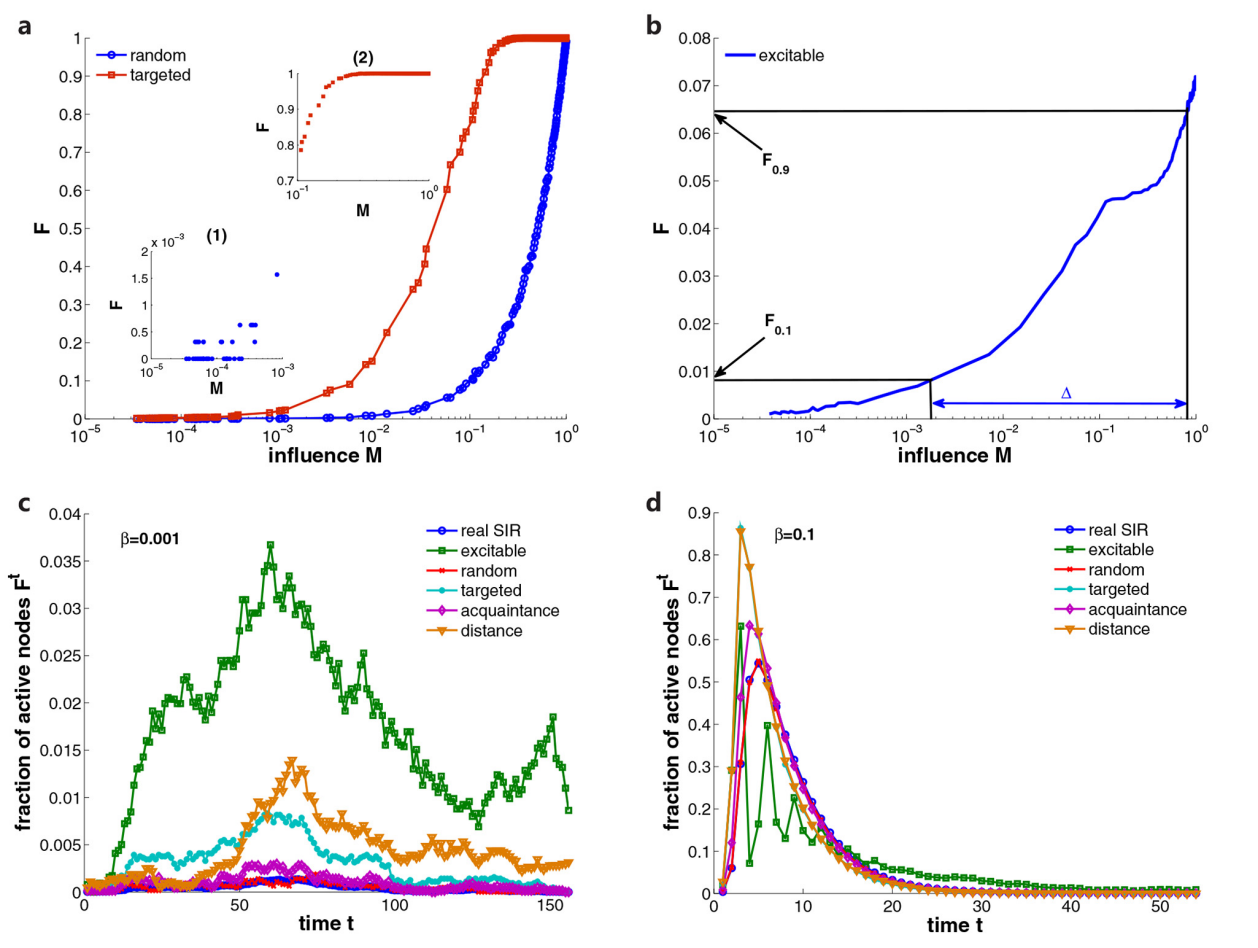
Response of sensor networks to SIR epidemic spreading. Here we run SIR model on facebook social network and select 10% of nodes as sensors. The average degree of sensor network is set as ⟨*k*⟩ = 4, so the coupling strength of excitable sensors is *s* = 0.25. We set *μ* = 0.2 in simulations. The source is selected as a hub with degree *k* = 1089. (a) The random sensors fail to detect small-scale epidemic spreading and targeted sensors saturate only after the spreading occupies about 20% of population. (b) The excitable sensor network is capable of detecting small-scale epidemic spreading and distinguishing large-scale spreading. Straight lines indicate relevant parameters to calculate the dynamic range Δ. (c) and (d) display the fraction of active sensors *F* ^*t*^ for different methods when we set *β* = 0.001 and *β* = 0.1 in SIR modeling respectively.

Fig 3(a) displays the responses of random and targeted sensors to SIR epidemic spreading. By varying the infection probability *β*, we can create diffusion instances with various influence. We select 10% of nodes in facebook social network as sensors. The response of random sensors follows a linear relationship with influence. In the range of small influence, random sensors fail to detect some small-scale spreading and, more importantly, cannot distinguish the influence clearly. This phenomenon is better illustrated in the inset of Fig 3(a). Many spreading processes have response *F* = 0, and large numbers of diffusion processes with distinct influence produce same response. In contrast to random strategy, targeted sensors perform better for small influence. The response is amplified because of the topological property of sensors, and the influence range that can trigger distinguishable responses is extended to the left by at least one order of magnitude. However, in the range of large influence, the response of targeted sensors saturates only when approximately 20% of individuals are infected. For spreading processes with influence larger than 20%, the response of targeted sensors is always 1. This dramatically diminishes the effective detecting range in which we can rank the influence correctly.

We now examine the performance of excitable sensor networks. In Fig 3(b), the response curve of the excitable sensor network is displayed. The sensors are capable of detecting small-scale epidemic spreading and distinguishing large-scale diffusion. This can be explained by Fig 3(c) and 3(d). In the case of small influence, we set the infection probability *β* = 0.001. Compared with other strategies, the fraction of active sensors *F* ^*t*^ for the excitable sensor network is greatly amplified because of the signal transmission among sensors. The random strategy reflects the real proportion of infected people at each time step. Targeted, distance and acquaintance strategies exploit sensors’ relatively central locations in the social network, thus enhancing the infected population of sensors. The excitable sensor network relies on both sensors’ topological advantages and signal transmission processes. In this way, small-scale spreading instances are more likely to be detected by the excitable sensor network. When the response is amplified, it makes the influence more distinguishable. For large-scale diffusion shown in Fig 3(d), where we set *β* = 0.1, a peak of infection appears. The fraction of active sensors for targeted strategy becomes the largest, which produces the early saturation of targeted sensors. In contrast, oscillations emerge in the excitable sensor network because of the self-inhibition effect. Once a large fraction of sensors are activated, they update into refractory state. Therefore, in the next time step, *F* ^*t*^ will drop dramatically. After these sensors changing back to resting state, they can be activated again and create another crest. This phenomenon prevents the early saturation of sensor networks. As a consequence, the excitable sensor network can correctly detect the influence of large-scale spreading.

**Fig 4:**
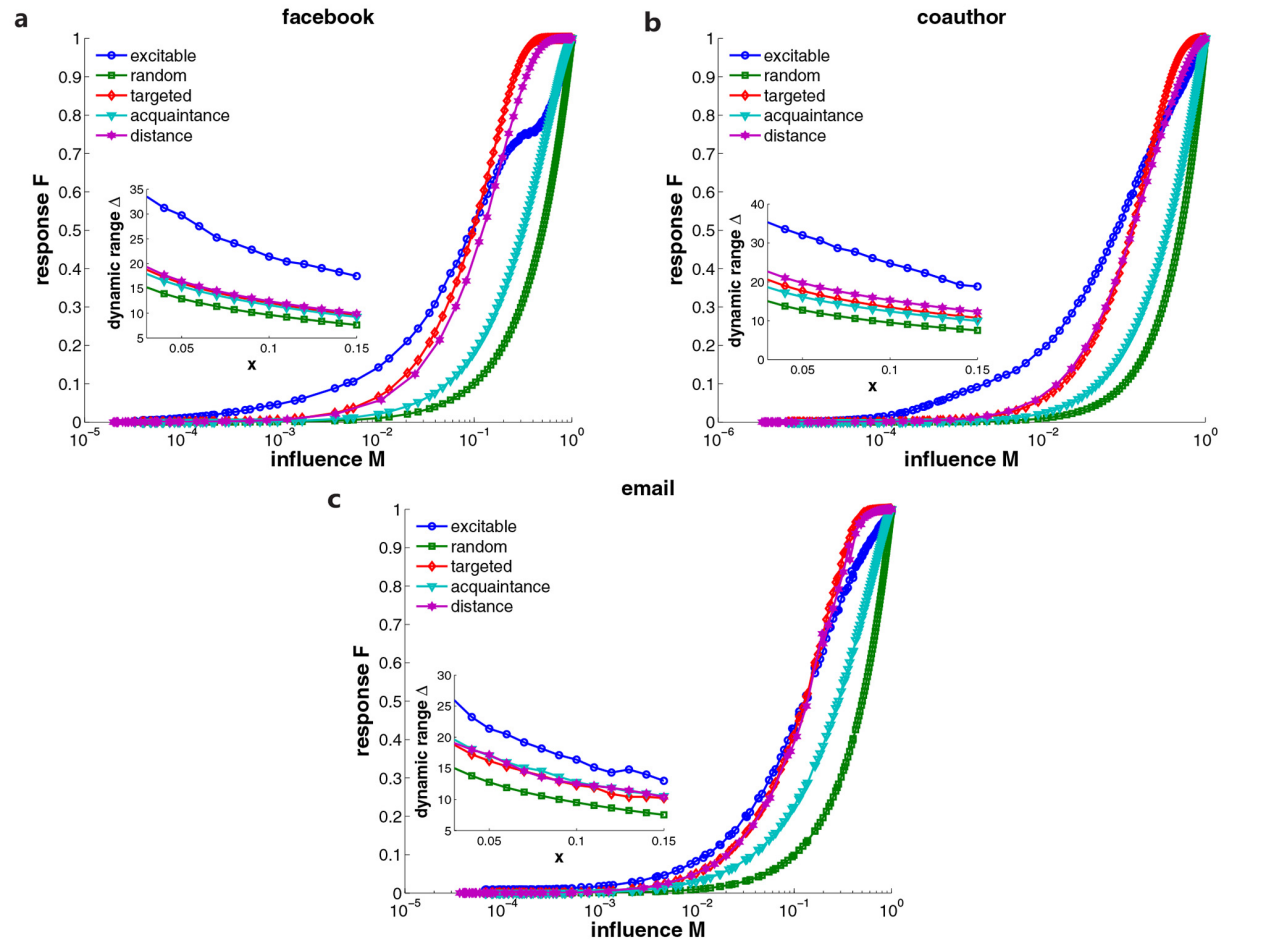
Comparison of performances of different strategies in response to SIR spreading dynamics. We apply SIR model on facebook (a), coauthor (b) and email (c) social networks, and display the response curve for each strategy. 10% of nodes are selected as sensors. We construct an excitable sensor network with average degree ⟨*k*⟩ = 4, and set *μ* = 0.2 in simulations. The sources are selected as hubs with degree *k* = 1089, 343 and 1383 respectively. The response curves for all cases are normalized to the unit interval [0, 1]. The insets show the dynamic range for each case when we vary the calculation interval [*F*_*x*_, *F*_1−*x*_] from *x* = 0.01 to *x* = 0.15.

To evaluate the results for other types of social networks, we also apply SIR model on coauthor and email social networks. Fig 4 displays the comparison of the performances of different strategies in response to SIR spreading dynamics for facebook, coauthor and email networks. In simulations, we select 10% of nodes as sensors and choose hubs as diffusion sources. We construct an excitable sensor network with average degree ⟨*k*⟩ = 4, and set *μ* = 0.2. To compare the response curves directly, we normalize the response for each strategy to the unit interval [0, 1]. For all considered social networks, the response curves share common properties: in the range of small influence, the response of excitable sensors stays larger than those of other methods, while it is suppressed for large-scale diffusion. This leads to a broader effective detection range in which spreading processes can be detected and ranked reliably. We calculate the dynamic range for each case by varying the interval [*F*_*x*_, *F*_1−*x*_] from *x* = 0.01 to *x* = 0.15. The insets show that the dynamic range of the excitable sensor network is considerably higher than those of random, targeted, acquaintance and distance strategies. This fact directly indicates the better performance of excitable sensor networks in detecting the influence of SIR spreading. In addition, random sensors perform worst because they are incapable of amplifying small-scale spreading.

Although we have tested the efficacy of the excitable sensor network for SIR spreading dynamics, it is still desirable to evaluate its performance for other spreading mechanisms. In S1 File, we perform SIS, Rumor and SIRL dynamics on facebook, coauthor and email social networks, obtaining similar results in Figures B-D in S1 File. All evidence supports that the excitable sensor network outperforms random, targeted, acquaintance and distance strategies. A general conclusion we obtain from the analysis is that the dynamic range of the excitable sensor network is higher for all considered spreading dynamics (SIR, SIS, Rumor and SIRL) and social networks (facebook, coauthor and email networks). The consistency of the results stems from the inherent property of excitable media. According to the dynamics of excitable elements, excitable networks can amplify weak stimuli and suppress intense one as well. This functional characteristic is independent of which spreading dynamics or social networks we adopt. Consequently, it is potentially applicable to various spreading dynamics and topological structures.

**Fig 5:**
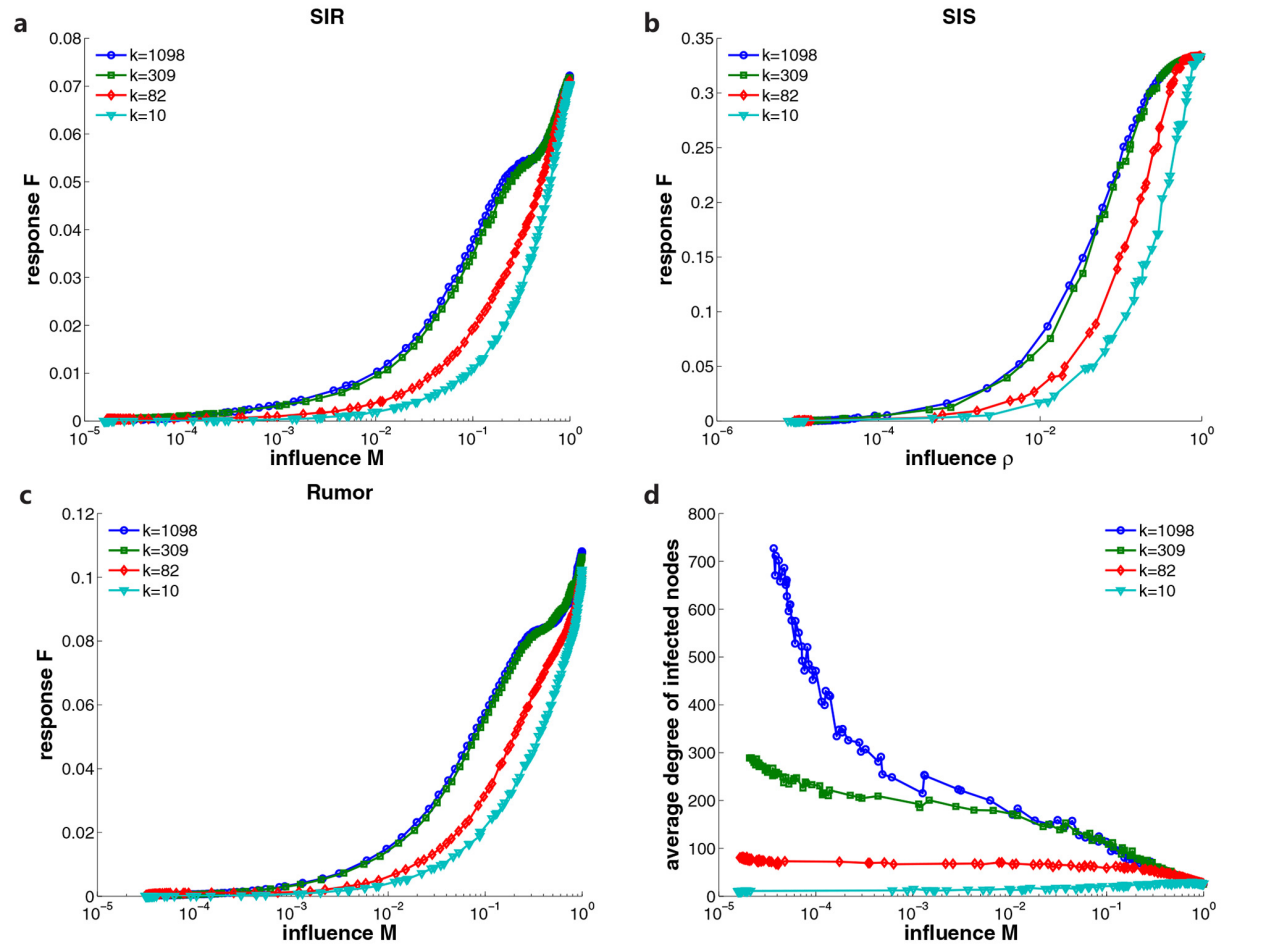
Response curves of excitable sensor networks for different spreading sources. The SIR (a), SIS (b) and Rumor (c) spreading models are applied on facebook social network. Four distinct nodes are selected as diffusion sources. The selected sources have degree *k* = 1089, 309, 82 and 10. The relationship between the response and spreading influence is presented. In (d) we plot the average degree of infected people versus the spreading influence for SIR model originating from different sources.

After we have verified the effectiveness of excitable sensor networks for various spreading models and social networks, we evaluate how excitable sensors respond to spreading processes originating from different sources. We select four distinct nodes in facebook social network as spreading origins. The selected nodes have degrees *k* = 1089, 309, 82 and 10, representing distinct groups of users. We run SIR, SIS and Rumor models on facebook social network originating from these sources. The relationship between the responses of excitable sensor networks and spreading influence is presented in Fig 5. Obviously, the response curves for sources with smaller degrees stay lower. The amplifying effect of excitable sensors is more effective for hubs in the region of small influence and decreases as the degree of source diminishes. The disparity in the amplifying effect can be explained by the property of infected nodes in spreading dynamics. Despite the fact that the spreading processes have same number of infected people, the property of these individuals varies significantly for different sources. In Fig 5(d), we plot the average degree of infected individuals versus spreading influence for SIR model originating from four selected spreading sources. Clearly, in the region of small influence, the average degree of infected people for a well-connected source is far higher than that for a low-degree source. As the spreading influence increases, this difference is narrowed because more nodes are infected. Fig 5(d) indicates that for a spreading process originating from a high-degree source, the infected people are also inclined to possess large numbers of connections. This will cause more sensors to be activated because we pick highly connected nodes as sensors. In this way, the response for a high-degree source will become higher. Because of this effect, excitable sensor networks are more likely to detect spreading instances from high-degree sources under same circumstances.

### Effect of the construction method of excitable sensor networks

After examining the performance of excitable sensor networks through simulations, we would like to discuss the impact of the construction method of sensor networks. First, we explain why the sensor network is constructed as a homogeneous random network. It has been found that homogeneous networks enhance the dynamic range more than heterogeneous networks [24, 25]. In ref [25], the dynamic range at critical state Δ can be predicted by

$$\begin{array}{c} \Delta =10\log_{10}\frac{2}{3F_{*}^{2}}-10\log_{10}\frac{\left\langle vu^{2}\right\rangle }{\left\langle v\right\rangle {\left\langle u\right\rangle }^{2}}, \end{array}$$ (8)

where F__*__ is the lower threshold response, *u* and *v* are the right and left dominant eigenvectors of sensor network’s adjacency matrix. Here $\langle vu^{2}\rangle =\sum_{i=1}^{i=N_{s}}v_{i}u_{i}^{2}/N_{s}$, $\langle v\rangle =\sum_{i=1}^{i=N_{s}}v_{i}/N_{s}$, and $\langle u\rangle =\sum_{i=1}^{i=N_{s}}u_{i}/N_{s}$. Given a fixed lower threshold response F__*__, only the term −10log_10_(⟨*vu* ^2^⟩/(⟨*v*⟩⟨*u*⟩^2^)) can affect the dynamic range Δ. In our case of undirected networks where *u*_*i*_ = *v*_*i*_, the second term becomes −10log_10_(⟨*u* ^3^⟩/⟨*u*⟩^3^). Since the entries of the dominant eigenvector are first-order approximations to the degrees of corresponding nodes (*u*_*i*_ ≈ *k*_*i*_) [25], the second term suggests that Δ should increase as the degree distribution becomes more homogeneous.

In addition to theoretical analysis, we also compare the performances of ER random and BA scale-free sensor networks through simulations in Supporting Information S1 File. In facebook social network, we select 10% of nodes as sensors and construct ER and BA networks with same average degree ⟨*k*⟩ = 10. The coupling strength *s* is adjusted to achieve the critical state for both cases. Results in Figure E in S1 File indicate that for all considered spreading dynamics, ER sensor networks have higher dynamic ranges consistently, which justifies our choice of homogenous structure of sensor networks.

Apart from the impact of network structure, we also need to check the effect of number of sensors, or the fraction of sensors *f*. We conduct a sensitivity analysis on the number of sensors. Specifically, we run SIR, SIS, Rumor and SIRL models on facebook social network for *f* ranging from 0.01 to 0.1. For all spreading dynamics, the shape of response curves does not dramatically change along with the number of sensors (see Figure F in S1 File). Meanwhile, the dynamic ranges almost remain unchanged for different fractions of sensors *f*. This indicates that the choice of sensor numbers would not affect our result significantly.

### Validation by real diffusion data

While we have verified the effectiveness of excitable sensor networks for diverse spreading models, it still remains unknown how our method performs for real-world spreading instances. There is evidence showing that the spreading processes in reality cannot be fully characterized by theoretical models [50]. Additionally, the spreading dynamics in social networks are also greatly affected by other human-related factors, such as homophily [51, 52], activity [53, 54], social reinforcement [55], and social influence bias [56]. Under these circumstances, many researchers turn to explore empirical diffusion data in various platforms. Here, we investigate some empirical spreading instances from Twitter [57], an online social networking and microblogging service that has gained worldwide popularity. We intend to validate the efficacy of excitable sensors using real-life information spreading in Twitter. Details about the Twitter data can be found in the Materials and Methods section.

**Fig 6:**
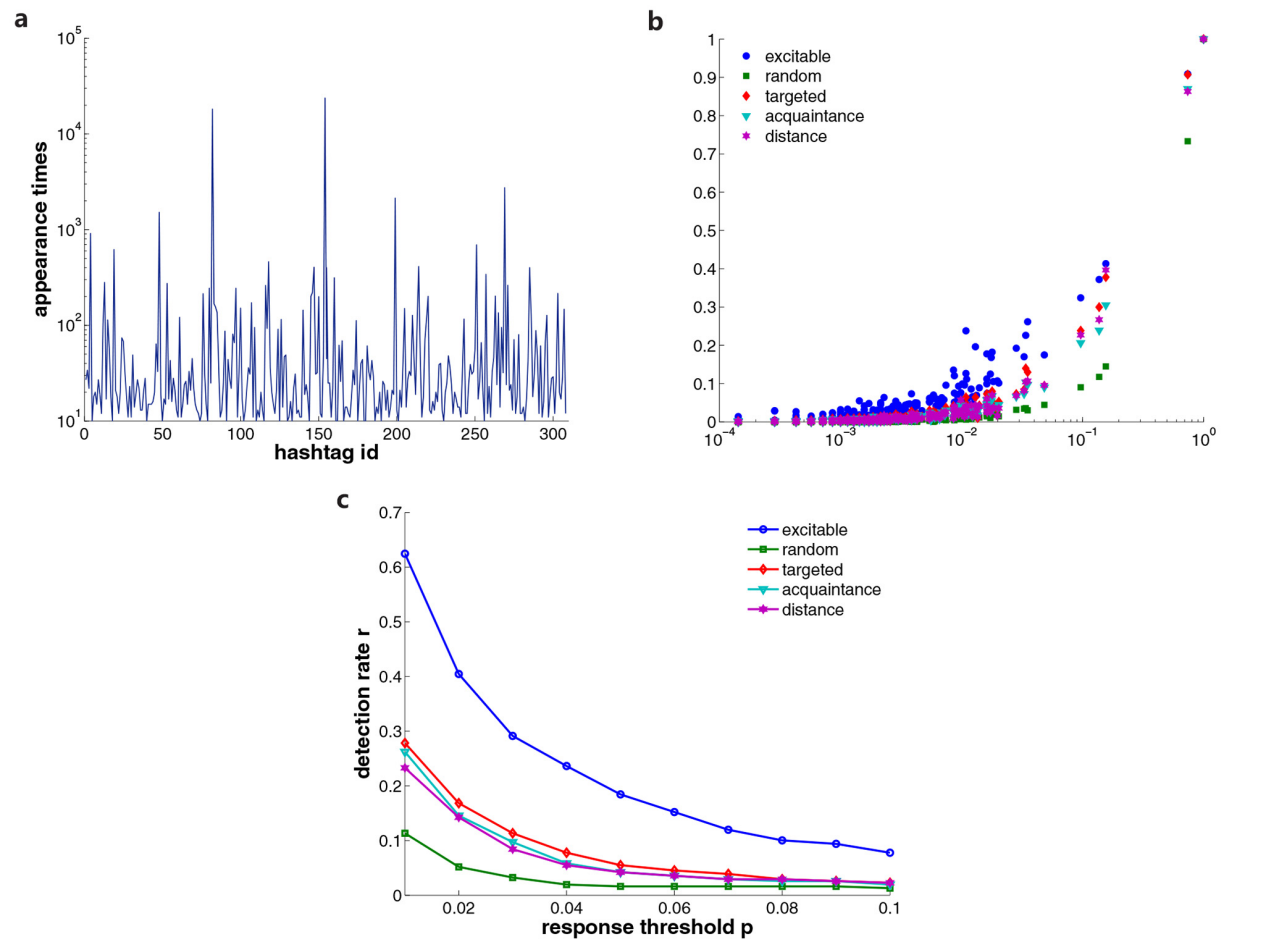
Validation of real diffusion instances in Twitter. The appearance frequency of selected 309 hashtags is shown in (a). Hashtag ids are ranked chronologically. In (b) we present the normalized response of different detecting strategies to these hashtags. We also display the detection rate *r* (the fraction of detected hashtags) if we consider a topic is detected only when the response is above a threshold *F*_*p*_ in (c).

To extract the information diffusion in Twitter, we examine the contents of tweets. In Twitter, users usually include hashtags (a word or an unspaced phrase prefixed with the number sign “#”) in their tweets when they refer to specific topics. Therefore, tracking the appearance of hashtags is a reliable method to infer the information diffusion in Twitter. To be concrete, we examine the tweets of 7014 users who have participated in discussions about the protest in Egypt on January 25th, 2011. During a time window of 17 days, we filter 309 distinct hashtags appearing more than 10 times among these users. We put a cutoff to omit small personal discussions that rarely diffuse among users. The frequency of these hashtags’ appearing is displayed in Fig 6(a), where hashtag ids are ranked chronologically. The intensity of hashtags spans several orders of magnitude. This property is suitable for testing the performance of sensors in response to spreading instances that vary by several orders of magnitude in influence.

To deploy sensors, we also require the structure of a social network. In our case, we reconstruct the mention network of users from the tweets to approximate the social network in real life. In Twitter, mentions (“@username”) usually convey personal conversations between users. Thus, we expect that the tie strength of mentions is stronger than that of following relations [58]. We do not track the follower network because Twitter has imposed a strict limit on the access rate of Twitter API, where we can obtain the list of followers for a given user. Moreover, even though we can track the followers of these users, the network structure may have changed significantly since the time of data collection, and some users may have even closed their accounts in Twitter. Considering all these limitations, we choose to use the mention network, which can represent contemporary social relations during the observation.

In detecting the topics in Twitter, we select 10% of users as sensors according to different strategies and form a sensor network with average degree ⟨*k*⟩ = 4 for excitable sensors. We regard each time step as a one-day interval. The state of a user at each time step is determined by the content of his/her tweets. If a user posts at least one tweet containing a specific hashtag during one time step, we assume that this user is infected by this hashtag. Otherwise, we assume that he/she is uninfected. During each time step, in the excitable sensor network, sensors can be activated either by infection, or by active neighboring sensors with the probability of *s* = 0.25 independently. The evolution dynamics remain the same as the above simulations, but the spreading model in the underlying social network is replaced by real diffusion instances. Here, we note that how we construct the social network cannot affect the spreading processes. All the information we use is “at what time, who has published a specific hashtag”. This information is independent of the network topology. Therefore, our choice of social network can only affect the selection of sensors and will not strongly change our detection results.

Fig 6(b) shows the responses of different detection strategies. The influence of a hashtag is defined as the proportion of users who have ever posted the hashtag during the observation time. Each dot represents a response value for a specific hashtag. Instead of following a smooth curve, the response dots are scattered for all methods we consider. This phenomenon can be partially explained by the distinction of spreading sources. Another factor may be that multiple sources or independent spreaders exist in the diffusion of hashtags [59]. In spite of this, the response shows an increasing trend as the influence increases, which makes large-scale diffusion distinguishable from small-scale ones. More importantly, in the range of small influence, the response of the excitable sensor network is higher on the whole. These results are in accordance with our prediction through simulations.

To quantify the performance of each method, we define the detection rate *r* to measure how many spreading instances a method can detect. Given a value *p* ∈ [0, 1], the detection rate *r*(*p*) is defined as the proportion of spreading instances whose responses are larger than *F*_*p*_, where *F*_*p*_ = *F*_0_ + *p*(*F*_*max*_ − *F*_0_). In other words, if we assume that a topic is detected only when the response is above *F*_*p*_, the detection rate *r*(*p*) quantifies the fraction of topics that sensors can detect. A higher detection rate means a better performance in detecting spreading. We present the detection rate *r* for *p* ∈ [0.01, 0.1] in Fig 6(d). Obviously, the detection rate of the excitable sensor network is the highest among all the methods. This indicates that excitable sensor networks are capable of detecting more spreading hashtags in real diffusion in Twitter.

## Discussion

The detection of spreading influence in social networks is an important issue both in theory and practice. Inspired by the mechanism by which the physical sensations of external stimuli emerge, we propose a method to construct excitable sensor networks in social networks. We study the dynamics of excitable sensors analytically, and find the relationship between the response and spreading influence. To compare the performance of our method with other sensor placement strategies, we conduct SIR, SIS, Rumor and SIRL simulations on three typical social networks. Because the nonlinear amplification property of excitable elements is independent of spreading dynamics, we obtain consistent conclusions: under the same circumstances, excitable sensor networks exhibit larger dynamic ranges, which implies that our method can react to spreading processes in a wider range of influence. Excitable sensor networks can not only detect small-scale spreading processes but also better distinguish large-scale diffusions. In addition, if the spreading processes originate from different sources, the average degree of infected nodes is higher for high-degree origins. Because of this fact, the excitable sensor network is more likely to detect spreading instances originating from well-connected sources. Moreover, we also validate the effectiveness of excitable sensor networks with real diffusion data in Twitter. The detection result shows that our method is capable of detecting more spreading topics. Our approach provides a new route for designing effective detection strategies.

## Supporting Information

S1 File Additional analysis of the dynamics and performance of excitable sensor networks. (PDF)
