# Supplementary material for: Detecting the Influence of Spreading in Social Networks with Excitable Sensor Networks
Source: PLoS One. 2015 May 7;10(5):e0124848. doi: 10.1371/journal.pone.0124848 (PMC4423969; doi:10.1371/journal.pone.0124848)
Supplement: S1 File — (PDF) [file pone.0124848.s001.pdf]

Detecting the Influence of Spreading in Social Networks with Excitable Sensor Networks

Sen Pei[1,2], Shaoting Tang[1,2,*], Zhiming Zheng[1,2,†]

1 School of Mathematics and Systems Science, Beihang University, Beijing, China
2 Laboratory of Mathematics, Informatics and Behavioral Semantics, Ministry of Education, Beijing, China

*** tangshaoting@buaa.edu.cn †zzheng@pku.edu.cn**

1 Dynamics of excitable sensors in heterogeneous networks

In the case of heterogeneous networks, we need to calculate I_i^t for each sensor i. Since I_i^t is significantly affected by sensor i's topological feature, we have to use the individual-level evolution equations rather than the mean-field approximation. Assuming the adjacency matrix of the social network is $\bar{A} = \{\bar{A}_{ij}\}_{N\times N}$, we give the evolution differential equations for each spreading model as follows.

SIR model For each node i in the social network, denote the probability of node i in susceptible, infected and recovered state at time t as $S_i(t)$, $I_i(t)$ and $R_i(t)$ respectively. The state updating differential equations read

$$\begin{aligned}
\frac{dI_i(t)}{dt} &= [1-\prod_{j=1}^{N}(1-\beta\bar{A}_{ij}I_j(t)S_i(t))] - \mu I_i(t) \approx \beta S_i(t)\sum_{j=1}^{N}\bar{A}_{ij}I_j(t) - \mu I_i(t),\\
\frac{dS_i(t)}{dt} &= -[1-\prod_{j=1}^{N}(1-\beta\bar{A}_{ij}I_j(t)S_i(t))] \approx -\beta S_i(t)\sum_{j=1}^{N}\bar{A}_{ij}I_j(t),\\
\frac{dR_i(t)}{dt} &= \mu I_i(t).
\end{aligned}$$

SIS model Denote the probability of node i in infected state at time t as $I_i(t)$. The evolution of $I_i(t)$ follows

$$\frac{dI_i(t)}{dt} = [1-\prod_{j=1}^{N}(1-\beta\bar{A}_{ij}I_j(t)(1-I_i(t)))] - \mu I_i(t) \approx \beta(1-I_i(t))\sum_{j=1}^{N}\bar{A}_{ij}I_j(t) - \mu I_i(t).$$

Rumor model Assume the probability of node i in the state of spreader, ignorant and stifler at time t as $S_i(t)$, $I_i(t)$ and $R_i(t)$ respectively. The evolution differential equations are

$$\begin{aligned}
\frac{dI_i(t)}{dt} &= -[1-\prod_{j=1}^{N}(1-\beta\bar{A}_{ij}S_j(t)I_i(t))] \approx -\beta I_i(t)\sum_{j=1}^{N}\bar{A}_{ij}S_j(t),\\
\frac{dS_i(t)}{dt} &= [1-\prod_{j=1}^{N}(1-\beta\bar{A}_{ij}S_j(t)I_i(t))] - [1-\prod_{j=1}^{N}(1-\mu\bar{A}_{ij}(S_j(t)+R_j(t))S_i(t))]\\
&\approx \beta I_i(t)\sum_{j=1}^{N}\bar{A}_{ij}S_j(t) - \mu S_i(t)\sum_{j=1}^{N}\bar{A}_{ij}(S_j(t)+R_j(t)),\\
\frac{dR_i(t)}{dt} &= [1-\prod_{j=1}^{N}(1-\mu\bar{A}_{ij}(S_j(t)+R_j(t))S_i(t))] \approx \mu S_i(t)\sum_{j=1}^{N}\bar{A}_{ij}(S_j(t)+R_j(t)).
\end{aligned}$$

SIRL model Denote the probability of node i in susceptible, infected and recovered state at time t as $S_i(t)$, $I_i(t)$ and $R_i(t)$. We have

$$\frac{dI_i(t)}{dt} = [1 - \prod_{j=1}^{N}(1 - \beta \bar{A}_{ij}\frac{L}{k_j}I_j(t)S_i(t))] - \mu I_i(t) \approx \beta L S_i(t)\sum_{j=1}^{N}\bar{A}_{ij}\frac{I_j(t)}{k_j} - \mu I_i(t),$$

$$\frac{dS_i(t)}{dt} = -[1 - \prod_{j=1}^{N}(1 - \beta \bar{A}_{ij}\frac{L}{k_j}I_j(t)S_i(t))] \approx -\beta L S_i(t)\sum_{j=1}^{N}\bar{A}_{ij}\frac{I_j(t)}{k_j},$$

$$\frac{dR_i(t)}{dt} = \mu I_i(t).$$

Here k_j represents the degree of node j.

Combining the above equations and Eq.1 in the main text, we can calculate the theoretical values of the response and influence for different infection rates β. In order to validate the theoretical analysis, we perform simulations on heterogeneous networks. In particular, we generate BA scale-free networks with size 10^5 and average degree 10, and run each spreading model to obtain simulation results. The relationship between the theoretical lines and simulation results is displayed in Figure A in S1 File. For all considered spreading dynamics, the theoretical lines agree well with the simulation results.

2 Performance of excitable sensor networks for various spreading dynamics

Although we have tested the efficacy of the excitable sensor network for SIR spreading dynamics in the main text, it is still desirable to evaluate its performance for other spreading mechanisms. To achieve this, we perform SIS, Rumor and SIRL dynamics on facebook, coauthor and email social networks, conducting similar analyses in Figures B-D in S1 File. Without loss of generality, we construct excitable sensor networks with average degree $\langle k \rangle = 4$. We set $\mu = 0.2$ for SIS and SIRL models and $\mu = 1$ for Rumor model. The contacting ability L in SIRL model is set to be 5. All simulations support that the excitable sensor network outperforms random, targeted, acquaintance and distance strategies.

3 Effect of the construction method of excitable sensor networks

To explore the impact of the topology of sensor networks, we run SIR, SIS, Rumor and SIRL models with both ER random and BA scale-free sensor networks. In facebook social network, we select 10% nodes as sensors and construct ER and BA networks with the same average degree $\langle k \rangle = 10$. The coupling strength s is adjusted to achieve the critical state for both cases. For each BA scale-free sensor network, we first calculate the eigenvalue of the adjacency matrix λ, and then set the coupling strength $s = 1/\lambda$. Results in Figure E in SI File indicate that ER sensor networks have higher dynamic ranges.

In order to check the effect of f, we conduct a sensitivity analysis on the number of sensors. We simulate SIR, SIS, Rumor and SIRL models on facebook social networks for f ranging from 0.01 to 0.1. In Figure F in S1 File, the shape of response curves is not dramatically changed by the number of sensors. At the same time, the dynamic ranges almost remain unchanged for different fractions of sensors f. This indicates that the choice of sensor numbers would not affect our result significantly.

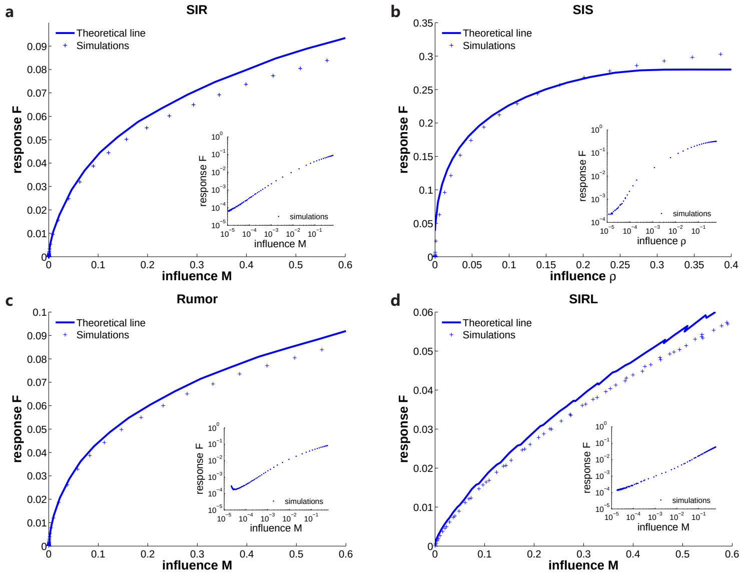

Figure A. Theoretical analysis of the dynamics of excitable sensors in heterogeneous networks. For SIR, SIS, Rumor and SIRL models, we display the relationship between the response and influence in (a), (b), (c) and (d) respectively. We adopt BA scale networks with size 10^5 and average degree 10. 10% of nodes are randomly selected to be sensors, which are connected in a homogeneous random network with average degree $\langle k \rangle = 10$. Solid lines are theoretical predictions and cross symbols represent simulation values. In simulations, we vary infection rate β and keep $\mu = 1$ for all models. The contacting ability L in SIRL model is set to be 5.

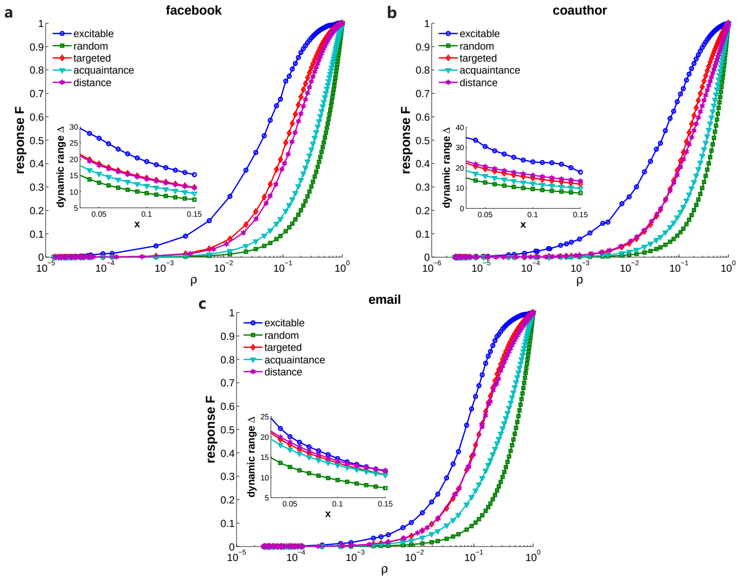

Figure B. Response to SIS spreading dynamics of each method. We run SIS model and display the normalized response curves for facebook (a), coauthor (b) and email (c) social networks. We select 10% of nodes as sensors. The sources are selected as hubs with degree $k = 1089$, 343 and 1383 respectively. The average degree of excitable sensor networks is $\langle k \rangle = 4$, and $\mu = 0.2$. The insets show the dynamic range for each case when we vary the calculation interval $[F_x, F_{1-x}]$ from $x = 0.01$ to $x = 0.15$.

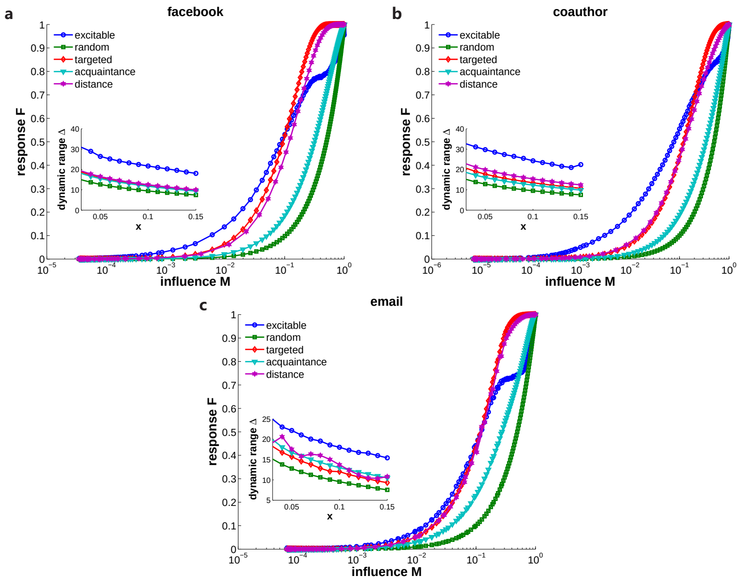

Figure C. Performances of different strategies in response to Rumor dynamics. The response curves for facebook (a), coauthor (b) and email (c) social networks are shown. We adopt Rumor dynamics and select source nodes with degree $k = 1089$, 343 and 1383 respectively. Response curves are normalized to the unit interval $[0, 1]$. The excitable sensor networks contain 10% of nodes, and have average degree $\langle k \rangle = 4$. The parameter μ is set to be 1. The dynamic range for each case is shown, where we vary the calculation interval $[F_x, F_{1-x}]$ from $x = 0.01$ to $x = 0.15$.

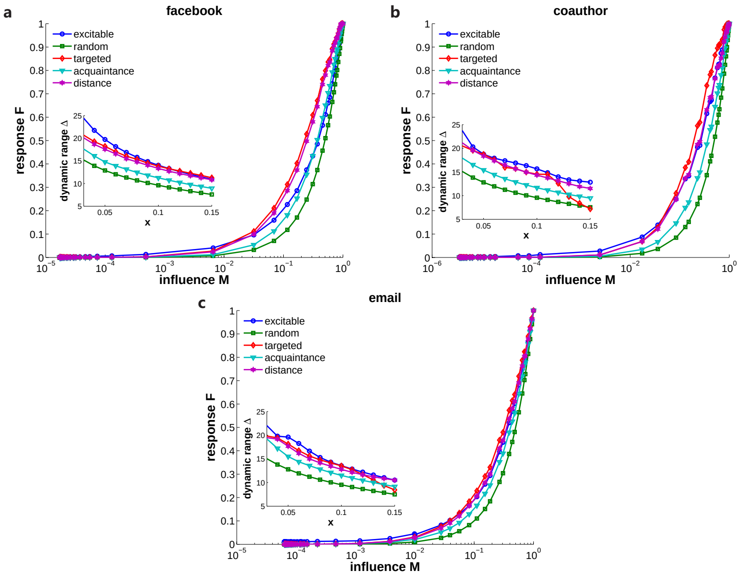

Figure D. Comparison of performances of different strategies in response to SIRL spreading dynamics. We apply SIRL model on facebook (a), coauthor (b) and email (c) social networks, and display the relationship between the response and spreading influence. 10% of nodes are selected as sensors. We construct excitable sensor networks with average degree $\langle k \rangle = 4$, and set $\mu = 0.2$, $L = 5$ in simulations. The sources are selected as hubs with degree $k = 1089$, 343 and 1383 respectively. The response curves for all cases are normalized to the unit interval. The insets show the dynamic range for each case when we vary the calculation interval $[F_x, F_{1-x}]$ from $x = 0.01$ to $x = 0.15$.

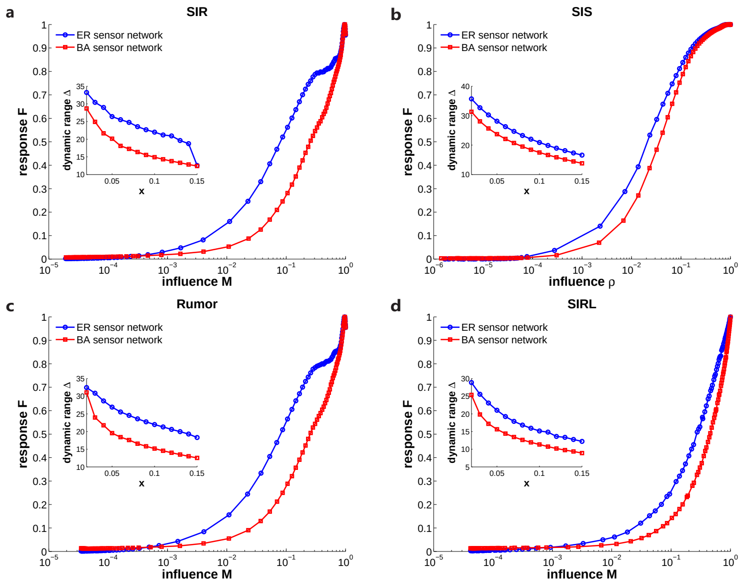

Figure E. Impact of the topology of excitable sensor networks. For facebook social network, we run SIR (a), SIS (b), Rumor (c) and SIRL (d) models with both ER random and BA scale-free sensor networks. The relationship between influence and response is displayed. The excitable sensor networks contain 10% nodes and have average degree $\langle k \rangle = 10$. We set $\mu = 1$ for all models and $L = 5$ for SIRL model. The source is selected as the hub with degree $k = 1089$. The insets show the dynamic ranges when we vary the calculation interval $[F_x, F_{1-x}]$ from $x = 0.01$ to $x = 0.15$.

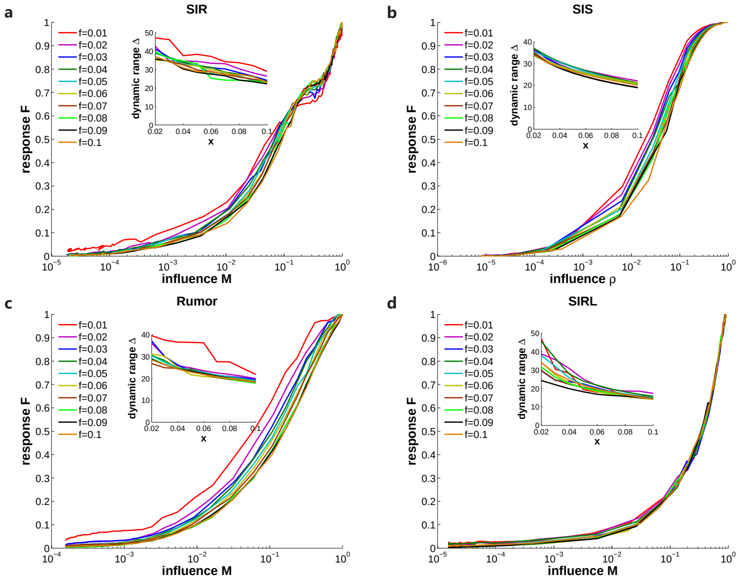

Figure F. Effect of the number of sensors. We run SIR (a), SIS (b), Rumor (c) and SIRL (d) models on facebook social network for f ranging from 0.01 to 0.1. The relationship between response and influence is displayed. The excitable sensor networks have average degree $\langle k \rangle = 4$. We set $\mu = 1$ for all models and $L = 5$ for SIRL model. The source is selected as the hub with degree $k = 1089$. The insets show the dynamic range for each case when we vary the calculation interval $[F_x, F_{1-x}]$ from $x = 0.01$ to $x = 0.15$.
